# Manipulating resident microbiota to enhance regulatory immune function to treat inflammatory bowel diseases

**DOI:** 10.1007/s00535-019-01618-1

**Published:** 2019-09-03

**Authors:** Yoshiyuki Mishima, Ryan Balfour Sartor

**Affiliations:** 1 Department of Internal Medicine II, Faculty of Medicine, Shimane University, Izumo, Shimane Japan; 2 Center for Gastrointestinal Biology and Disease, Division of Gastroenterology and Hepatology, Department of Medicine, University of North Carolina, Room 7309A MBRB, CB# 7032, Chapel Hill, NC 27599-7032 USA

**Keywords:** Crohn’s disease, Ulcerative colitis, Treatment, Bacteria, Live biotherapeutic products

## Abstract

Altered intestinal microbial composition (dysbiosis) and metabolic products activate aggressive mucosal immune responses that mediate inflammatory bowel diseases (IBD). This dysbiosis impairs the function of regulatory immune cells, which normally promote mucosal homeostasis. Normalizing and maintaining regulatory immune cell function by correcting dysbiosis provides a promising approach to treat IBD patients. However, existing microbe-targeted therapies, including antibiotics, prebiotics, probiotics, and fecal microbial transplantation, provide variable outcomes that are not optimal for current clinical application. This review discusses recent progress in understanding the dysbiosis of IBD and the basis for therapeutic restoration of homeostatic immune function by manipulating an individual patient’s microbiota composition and function. We believe that identifying more precise therapeutic targets and developing appropriate rapid diagnostic tools will guide more effective and safer microbe-based induction and maintenance treatments for IBD patients that can be applied in a personalized manner.

## Background

**Fig. 1:**
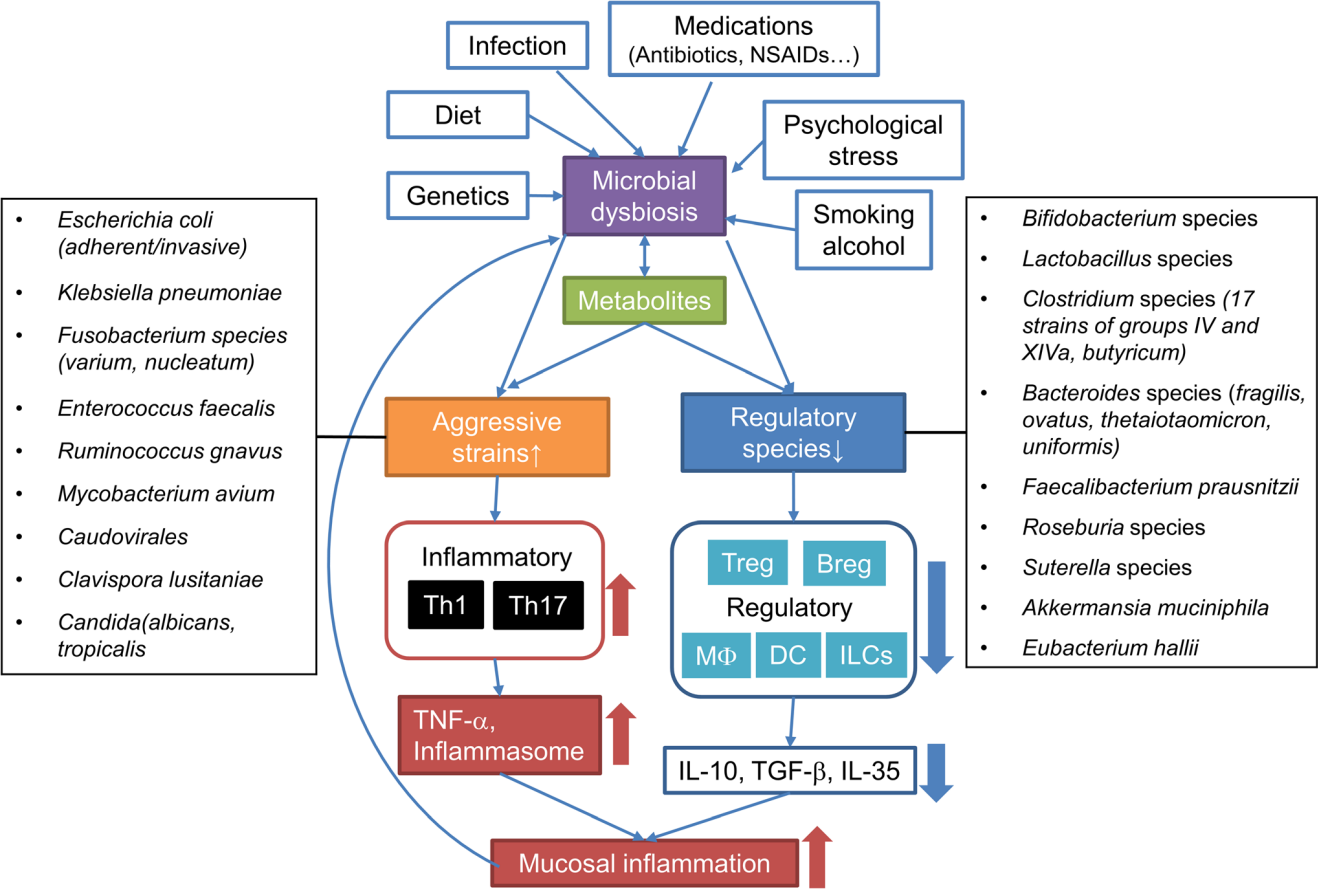
Dysbiosis-associated mucosal immune-dysfunction in IBD. Enteric infection, medications including antibiotics, NSAIDs and immunosuppressive drugs, diet, smoking and alcohol, psychological stress in susceptible genetic individuals cause microbial dysbiosis and metabolic changes. Prolonged dysbiotic conditions characterized by increased aggressive bacterial strains and decreased regulatory species lead to dysfunction of mucosal immune response. Aggressive microbial groups activate inflammatory response by inducing Th1/Th17-effector cells, while decreased regulatory species impair the induction and function of regulatory cells that include regulatory T cells (Treg), B cells (Breg), macrophages (MΦ), dendritic cells (DC) and innate lymphoid cells (ILCs). This imbalance of mucosal cytokine profiles in combination with defective barrier function sustains mucosal inflammation and can potentially lead to IBD in susceptible individuals

Hundreds of trillions of microorganisms, including bacteria, virus, fungi and archaea, reside in our distal intestines and mutually interact with co-evolved host immune cells in a beneficial reciprocal relationship that is influenced by host genetics and environmental factors, including the diet [1–3]. The microbiota evolved to colonize specialized ecological niches of the human gastrointestinal tract and to utilize variable diets, while the human mucosal immune system evolved to protect the host from harmful microbial pathogen exposures, yet prevent chronic intestinal inflammation [3, 4]. Enteric resident microbiota exists as a consortium that contains both putative proinflammatory and protective strains [5, 6]. A delicate balance between those functionally distinct populations is maintained in healthy individuals, while patients with inflammatory bowel diseases (IBD), including Crohn’s disease (CD) and ulcerative colitis (UC), harbor an altered gut microbial composition (dysbiosis) defined as increased potentially aggressive species in parallel with decreased anti-inflammatory groups [5–8]. Gut microbial diversity decreases and metabolic functions are altered in IBD patients, suggesting a loss of protective bacteria and their functions in IBD [9–11]. Prolonged dysbiotic conditions lead to dysfunction of the host immune system, which is considered the key mediator of the chronic inflammation of IBD [6, 11] (Fig. 1).

The activation, migration, proliferation, differentiation and maintenance of a variety of mucosal immune cells are directly regulated by resident microbiota. These activated immune cells cooperate to maintain intestinal homeostasis in normal hosts [4]. Inflammatory immune cells help eliminate invading pathogens by highly effective redundant innate and adaptive immune mechanisms. Microbiota boosts the innate immune response against pathogens by stimulating secretion of antimicrobial peptides and cytokines such as TNFα, IL-22 and IL-17, and activating the inflammasome for anti-pathogen defense [2]. On the other hand, regulatory immune cells including regulatory T cells [12–15], B cells [16–19], dendritic cells [20, 21], macrophages [22] and innate lymphoid cells (ILCs) [23] counteract excessive inflammatory reactions [2, 4, 5, 24–26]. The frequency and functions of these regulatory cells are impaired in IBD, but can potentially be stimulated by microbial manipulation to restore immune homeostasis to reverse and normalize dysregulated immune function and ameliorate mucosal inflammation [12, 27–29]. Thus, targeted induction and maintenance of regulatory immune cells by manipulating microbial profiles and functions offer a promising approach to treat IBD patients.

In this review, we discuss the characteristics of the dysbiosis associated with IBD to identify potential treatment targets as well as recent progress regarding therapeutic induction of regulatory immune cells by resident bacteria and their products. Understanding the detailed mechanisms of dysbiosis will open new insights into the pathogenesis of IBD and uncover new strategies to normalize regulatory immune cell functions by manipulating the microbiota as more physiologic and effective treatment options for IBD patients.

## Dysbiosis-associated mucosal immune-dysfunction in IBD

It is still unclear whether dysbiosis is a cause or consequence of inflammation in IBD patients [30]. Mucosal inflammation can directly alter microbial composition by increasing oxygen concentrations and metabolic changes that might expand colitogenic aerobic or facultative oxygen-tolerant anaerobic species [31, 32]. In addition, inflammation stimulates macrophages to produce nitrate oxide (NO) and increase the bacterial groups that can synthesize NO. Some colitogenic bacterial species utilize NO to regulate their membrane electron transport and protect from oxidative stress, which are potentially beneficial for their survival [33]. For example, parasite infection with *Toxoplasma gondii* induces mucosal inflammation and marked bacterial dysbiosis, which might be due to upregulated nitrate synthesis that serves as a source for anaerobic respiration and supports overgrowth of colitogenic *Enterobacteriaceae* [34]. Likewise, we demonstrated that the relative composition of a defined group of human IBD-relevant bacterial strains evolved as colitis progressed in selectively colonized gnotobiotic *Il10*^−*/*−^ mice in contrast to stable profiles of the same strains in identically colonized wild-type mice [35]. In parallel, experimental colonic inflammation alters luminal bacterial gene expression that potentially transforms certain resident bacterial species into a more colitogenic phenotype that can sustain inflammation [36–38].

On the other hand, dysbiosis can drive inflammatory immune responses. Dysbiosis was present in a significant portion of CD patients in a pediatric inception cohort at the time diagnosis [39] and multiple genetic polymorphisms associated with IBD, such as *NOD2, ATG16L1, CARD9, CLEC7A, HLAs* and mucin-related genes, influence the microbiome and its function [40, 41]. Genetically dysfunctional PPAR-γ signaling or elimination of PPAR-γ-producing bacteria with antibiotics promote a dysbiotic expansion of potentially colitogenic bacteria by reducing the bioavailability of respiratory electron acceptors [42]. In addition, exposure of antibiotics to IBD mothers during pregnancy increase risk of very early-onset IBD in their children, suggesting that antibiotic-mediated dysbiosis enhances the risk of mucosal inflammation in susceptible hosts [43]. In mouse studies, transfer of bacteria strains associated with IBD induces intestinal inflammation in susceptible gnotobiotic mice [35, 44] and fecal transplants from IBD donors to germ-free mice stimulate increased numbers of Th17 cells and inflammatory mediators compared with transfer of feces from healthy donors, which enhance numbers of inducible Tregs [45, 46]. These observations indicate that dysbiosis can have a causative role in inducing inflammation. Taken together, dysbiosis and intestinal inflammation appear to influence each other and synergistically perpetuate chronic immune activation that mediates IBD [47].

Since aggressive Th1 and Th17 immune responses directed against dysbiotic resident microbiota have a central role in the pathogenesis of CD [5, 6, 48], correcting dysbiosis can potentially alleviate gut inflammation and be an attractive therapeutic strategy for IBD. Effective microbial therapy depends on identifying the specific dominant microbial drivers of pathogenic effector immune responses. Multiple cohort studies in IBD patients and gnotobiotic animal experiments have identified specific bacterial families and species that influence dysbiosis-mediated immune dysfunction. Segmented filamentous bacteria (SFB) strongly activate Th17 responses in the small intestine of mice [49, 50], although SFB induces non-inflammatory homeostatic Th17 cells rather than infection-induced inflammatory Th17 [51]. Adherent-invasive *Escherichia coli* (AIEC) and *Citrobacter rodentium* induce colitogenic Th1 and Th17 responses in the colon [50, 52, 53] and oral *Klebsiella pneumoniae* strains activate Th1 cells and colitis when they ectopically colonize the colon [54].

Moreover, dysbiosis alters microbial metabolites in the intestine. For example, hydrogen sulfide, a dietary metabolite, is a toxin associated with progression of mucosal inflammation in UC by blocking butyrate metabolism in colonic epithelial cells [55]. Defective detoxification capacity may be involved in the pathogenesis of UC [55]. Trimethylamine-*N*-oxide (TMAO) is generated by enteric anaerobes through the digestion of dietary carnitine and phosphatidylcholine [56]. Western diets (enriched in fat, phosphatidylcholine, and l-carnitine) potentially promote mucosal inflammation through TMAO induction [57]. Both hydrogen sulfide and TMAO are increased in the feces of IBD patients [55, 56]. In contrast, multiple bacterial metabolites exert protective activities that stimulate mucosal homeostasis. For example, the bacterial metabolites short-chain fatty acids (SCFA), primarily butyrate and propionate, are the primary nutrients for colonic epithelial cells and stimulate regulatory T cells (Tregs) [58]. These protective metabolites are consistently decreased in IBD patients with dysbiosis [11]. Bacteroides-derived sphingolipids have defined protective roles in mucosal inflammation and are decreased in IBD subjects, although host-derived sphingolipids were identified as the most differentially abundant metabolites in stool from IBD patients [59]. Bile acids are also bacterial metabolites with pleotropic functions, including the regulation of metabolism and inflammation through interactions with both microbial and host receptors [60]. Ursodeoxycholic acid, a hydrophilic secondary bile acid, ameliorates mouse experimental colitis by expanding anti-inflammatory cluster XIVa *Clostridium* and *Akkermansia muciniphila* [61]. Dysbiosis in enteric bacteria changes bile acid receptor FXR expression, which is protective for experimental colitis models through inhibiting NF-kB signaling [62]. Conjugated bile acids activate sphingosine 1-phosphage receptor 2-mediated protective pathways [63]. Other protective bacterial metabolites, including indoles are decreased during dysbiosis [11, 64, 65], which subsequently decrease homeostatic immune cell and mucosal barrier functions. Indole metabolites stimulate IL-22 production by LP ILCs that mediate epithelial protection through AhR [66, 67].

Collectively, dysbiosis markedly impacts both inflammatory and regulatory responses of the host’s immune system. However, it is still unclear what types, degree and duration of dysbiosis are necessary to cause dysregulated mucosal immunity and IBD development and if and how rapidly correction of this dysbiosis can normalize homeostatic processes in IBD patients with genetic defects in immunoregulation and epithelial barrier function. It will be necessary to address these questions and more fully understand the IBD-specific dysbiosis to develop into new diagnostic tools and therapeutic targets for IBD.

## Resident bacteria activate regulatory immune cells and signaling pathways

The regulatory cytokine IL-10, together with TGF-β and IL-35, is a key mediator in microbe-mediated gut homeostasis [68] and plays a pivotal role in the pathogenesis of IBD [69]. Multiple genome-wide association studies showed that genetic polymorphisms in the IL-10 signaling pathway are associated with worsening phenotype of UC and early onset of CD [70–72]. However, subcutaneous supplementation of recombinant IL-10 protein once daily did not prevent postoperative recurrence in the CD patients and raised safety concerns [73–75]. This negative outcome might be due to an improper selection of the patients (especially those with high mucosal IL-10 level due to IL-10 signaling-related gene polymorphisms), or inappropriate dose, timing of treatment and delivery method of IL-10 [73]. Since IL-10 is a short-life cytokine and functions locally, a more physiological and safer strategy may be to stimulate regulatory immune cells to secret adequate IL-10 at the intestinal mucosal site. For example, replacing missing or decreased regulatory resident microbes [12] or genetically engineered IL-10-secreting bacteria [76, 77] may be effective strategies, because they do not require frequent administration of high dose of IL-10 in the circulation and should have low toxicity profiles.

Among the regulatory immune cells, regulatory T cells (Treg), consisting of thymus-derived Treg (tTreg) and inducible Treg (iTreg), have been most thoroughly studied in the pathogenesis of IBD [13–15]. iTreg are induced by normal resident bacteria but several studies showed that specific bacterial species are capable of inducing and maintaining iTreg. Human-derived 17 strains of *Clostridium* species induce Foxp3^+^CD4^+^Treg through IL-10, TGF-β1, butyrate and inducible T cell costimulatory (ICOS), and prevent mucosal inflammation in several murine colitis models [12, 78]. Polysaccharides from *B. fragilis* [79, 80] and protein components and supernatants of *F. prausnitzii* [81, 82] induce IL-10-producing mucosal Treg in mice. Recent studies show that a microbiota-activated iTreg population co-expressing RORγt^+^Foxp3^+^CD4^+^ (RORγt^+^Treg) possesses strong anti-inflammatory functions in the intestine [45, 83–85]. Normal resident bacteria as a whole are capable of inducing this population [19], but which bacterial strains or metabolites most efficiently activate RORγt^+^Tregs remain unknown. Britton et al. showed that transplant of feces from healthy human subjects induced higher concentrations of colonic lamina propria (LP) iTreg than did transfer of stools from IBD patients, which preferentially activated RORγt^+^ Th17 cells [45]. Bacterial metabolites can also induce Treg and intestinal homeostasis. Gut resident microbiota ferment dietary fiber to develop SCFAs that are essential in maintaining mucosal homeostasis. In particular, butyrate produced by the *Firmicutes* family members *Ruminococcaceae*, *Lachnospiraceae*, *Erysipelotrichaceae* or *Clostridiaceae,* activate and maintain intestinal Foxp3^+^ regulatory T cells. Treg induction by butyrate is mediated through activating G protein-coupled receptors (GPCRs), such as GPR41, GPR43, and GPR109A, and through inhibiting histone deacetylase synthesis [86, 87]. Another SCFA, propionate, also increases colonic Treg numbers by signaling via Ffar2 on Tregs and alleviates experimental colitis [58, 88]. Tryptophan is a major precursor of microbiota-derived AHR agonists such as various indoles that regulate chronic intestinal inflammation by promoting IL-10-producing T regulatory-1 (Tr1) cell induction and increasing the frequency of CD103^+^CD11b^−^ regulatory DCs [64–66].

Regulatory B cells (Breg) exert a prominent role in mucosal homeostasis by directly inhibiting inflammatory responses through the regulatory cytokines IL-10, TGF-β and IL-35 that enhance Treg function and expansion, and inhibit effector APC function [16–19, 89, 90]. We have shown that IL-10-producing immunoregulatory Breg are induced by resident bacteria through the TLR2/MyD88, Akt and PI3Kinase signaling pathway [19]. Identifying the resident microbiota families and species that preferentially activate Bregs would be helpful to develop new therapeutic reagents for IBD management. Based on our studies, it is likely that different bacterial populations, bacterial components and metabolites activate Bregs and Tregs, although some overlap may exist. For example, TLRs 2 and 9 ligands activate IL-10 production by B cells but not T cells [19].

Dendritic cells (DCs) classically act as APC and CX3CR1^intermediate^CD70^+^CD11b^+^ DCs in mouse or CD14^+^CD163^low^ DCs in humans can activate inflammatory Th17 cells [91, 92]. In contrast, DCs expressing CD103 have tolerogenic activities, promote iTreg differentiation, and inhibit intestinal Th1/Th17 immune responses by producing TGF-β, retinoic acid, AhR ligands and carbonic anhydrate I epitope peptide [26, 91, 93–95]. Resident bacteria also stimulate CX3CR1^+^CD103^−^CD11b^+^ DCs to augment the proliferation of Treg cells in an IFN-β and TLR4-dependent manner in mice [96].

Macrophages exhibit plasticity in their activation phenotype under different cytokine conditions [97, 98]. CD11c^high^CCR2^+^CX3CR1^+^ macrophages or M1 macrophages triggered by polarization signals from IFN-γ and microbial stimulation are proinflammatory [99]. Dysbiosis in IBD preferentially alters the dominant phenotype of intestinal LP macrophages into proinflammatory cells that exaggerate mucosal inflammation [100, 101]. Alternatively, CD11c^−^CCR2^−^CX3CR1^−^ macrophages or M2 macrophages induced by IL-4, IL-13, IL-10 or TGF-β show an anti-inflammatory profile in experimental colitis and are decreased in the colons of patients with active IBD [26, 99–101]. M2 macrophages are preferentially induced by a mixture of probiotics (Vivomixx) containing four strains of *Lactobacilli*, three strains of *Bifidobacteria*, and *Streptococcus thermophiles* [102]. GPBAR1, the primary and secondary bile acid receptor, on macrophages regulates the M1/M2 phenotype and alleviates murine colitis [103].

ILCs are innate cells that are induced by bacterial stimulation, including indoles that serve as AhR ligands, and influence intestinal homeostasis [104, 105]. Regulatory ILCs, defined as Lin^−^CD45^+^CD127^+^IL-10^+^ ILCs that are mainly located in the small intestinal LP can suppress the activation of proinflammatory ILC1s and ILC3s and confer protection from innate intestinal inflammation through secreting IL-10 and TGF-β1 [23]. However, further studies will be required to determine which type of bacterial stimulation specifically induces regulatory ILCs.

## Reversing dysbiosis as a nontoxic treatment of IBD

Recent progress in IBD treatments including the expansion of biological agents has provided rapid clinical remission and improved the quality of life in many IBD patients [106, 107]. However, those potent immunosuppressive therapies are not always effective, are quite expensive and potentially induce serious side effects [106, 107]. Recent reports reveal that the overall treatment efficacy, safety and cost-effectiveness of biologic therapies are not as striking as expected. For example, anti-TNF agents rapidly suppress inflammation and induce remission but did not change the long-term course in certain subsets of pediatric CD patients [108]. Murthy et al. showed that anti-TNF therapy did not reduce the frequency of hospitalization and surgical treatments in CD patients [109]. Therefore, more physiological approaches to induce and sustain remissions with limited toxicity and high cost-effectiveness are needed. Based on the current knowledge in the pathogenesis of IBD, manipulating the microbiota is considered as one of the rational treatment strategies for IBD [6, 28, 110, 111]. However, existing microbiota-targeting therapies including antibiotics, prebiotics, probiotics, and fecal microbial transplantation (FMT) demonstrate inconsistent results and the overall outcomes are not satisfactory in clinical practice [6, 28, 112].

Single antibiotic therapies provide modest effects to certain group of CD patients [113–116]. Oral metronidazole and ciprofloxacin are effective for anal lesions and delay of postoperative recurrence in CD [113, 115]. Rifaximin has a favorable safety profile because of the minimal systemic absorption, but does not yet have validated efficacy [114]. Broad spectrum antibiotics also can be effective in active IBD [113, 115, 117] and combination of antibiotics targeting *Fusobacterium varium* improved the outcome of UC patients [118]. Despite some favorable clinical effects, the long-term use of broad spectrum antibiotics potentially eliminates beneficial resident microbiota, induces antibiotic-resident species and creates a different type of dysbiosis by decreasing bacterial diversity [119].

Currently available probiotics potentially modulate dysbiosis in IBD, but their effects are transient and limited in most IBD subsets [6, 111, 112]. The possible reasons are: (1) single bacterial strains or combinations of traditional probiotics are not designed to replace the microbial species that are depleted in IBD patients and are unlikely to be effective given the broad heterogeneity in the microbial profile of individual IBD patients [9]. The microbiome profile of each IBD patient is different and defined IBD-specific dysbiosis is not present in all patients. (2) Most existing probiotics find colonization resistance in the host intestine, so that they do not colonize and are, therefore, present for a limited period, even after prolonged administration [120]. Baseline personalized host and mucosal microbial features are associated with probiotics persistence [121]. (3) The treatment timing is important to achieve the best effect of probiotics. (4) The proper delivery methods of live bacteria should be considered [122]. The most common species of current probiotics are *Lactobacillus* and *Bifidobacterium*, which have limited effects on IBD [112, 123]. Several clinical trials demonstrated that combination probiotics VSL#3 (containing 8 live bacterial species), *E. coli Nissle*, *B. bifidum* + *L. acidophilus*, *Lactobacillus GG* were effective in UC patients [112]. However, as mentioned above, the overall outcome of these probiotics is not fully satisfactory and new candidates for more effective colonizing probiotics that include combinations of protective resident strains (live biotherapeutic products, LBPs) are emerging [122].

Prebiotics, non-digestible carbohydrates that are metabolized by resident bacteria, can improve the composition and metabolic function of beneficial resident intestinal bacterial species [124]. Inulin, fructo-oligosaccharide, galacto-oligosaccharide, and lactulose are commonly used as prebiotics [124, 125]. Those prebiotics can increase the synthesis of SCFA, which improve barrier function, enhance regulatory immune responses and prevent pathobiont invasion by reducing pH levels in the intestine [125, 126]. In clinical practice, prebiotics can provide beneficial effect in IBD treatment, but their effects are modest with inconsistent results [28, 116, 125]. More mechanistic studies underlying the interactions among prebiotics, IBD-related dysbiosis and regulatory immune cells are required.

FMT is established as a standard treatment for recurrent *Clostridium difficile* infection, while the efficacy of FMT on IBD is still controversial [112, 127, 128]. Initial clinical studies show that FMT is effective at inducing remission in a small subset of UC patients with variable results in different studies; this variability might be due to different experimental designs including donor selection, delivery methods, pre-transplant preparation, frequency and timing of administration, as well as suitable controls. Recently, a randomized placebo-controlled trial demonstrated that multidonor intensive FMT with repeat administration 5 times/week for 6 weeks improved the efficacy in treating active UC [129]. Achieving remission by FMT was associated with increased microbial diversity with enrichment of *Eubacterium hallii* and *Roseburia inulivorans,* and increased levels of SCFA biosynthesis and secondary bile acids in the patients’ stool [130]. These types of deep analyses of microbial and host predictors of success vs failure will identify the characteristics of optimal donors and recipients to guide future application of this approach to treating IBD patients. However, importantly, FMT potentially can cause life-threatening side effects. The Food and Drug Administration (FDA) recently issued an alert after 2 patients died after FMT due to multi-antibiotics-resistant bacterial infection. These issues should be carefully considered in clinical practice.

The use of various combinations of specific intestinal-protective microbial strains or their metabolites may be safer and potentially more effective than whole FMT. This approach is more likely to achieve regulatory approval and be amenable to treating individual patients by matching replacement therapies in a rational targeted fashion based on the individual’s profile of fecal bacteria and metabolites (personalized therapy). The next generation of LBP or microbial products may include *Faecalibacterium prausnitzii*, which has a high capacity to induce IL-10-producing Treg [81, 82], *Clostridium* species (17 strains of clusters IV and XIVa, *C. butyricum*) [12, 78, 131] and *Bacteroides* species (*thetaiotaomicron* [132], *uniformis* [133], *ovatus* [134], and *fragilis* [79, 80]). Many other novel LBP formulations are being developed to replace protective bacterial species or homeostatic microbial products that are decreased in IBD patients.

## Future treatment options in IBD

Due to the increasing demand of microbe-based therapeutics, multiple preclinical and clinical trials are currently underway or planned to find better treatment approach in IBD [111]. Several novel strategies that extend beyond traditional antimicrobial and LBP approaches are briefly mentioned. These include targeting specific pathobionts and modifying bacterial functions by genetic engineering or pharmacologic approaches. Strategies to directly modulate specific pathobionts include preventing AIEC mucosal attachment by blocking fimH [135], depletion of pathobionts with bacteriophages [136], CRISPER-CAS editing to produce specific bacteriocines [137] and replacing ecologic niches with competing commensals [6, 111]. Despite remaining safety and environmental concerns, genetically modified bacteria such as anti-TNF nanobody-producing *Lactobacillus* species [138, IL-35 producing *E. coli* [139], and IL-10-, IL-27-, HO-1-secreting *Lactococcus* species could more efficiently alleviate mucosal inflammation by promoting a homeostatic immunologic profile, including Treg induction [90, 111, 140]. Moreover, a 16-kDa protein of helminths produced in *E. coli* protects against DSS-induced colitis by inhibiting PPAR-α signaling [141] and the formulation of several strains with beneficial other additives have been proposed [111]. Precision editing of the gut microbiota by tungstate ameliorates experimental gut inflammation through preventing the dysbiotic expansion of *Enterobacteriaceae* [142]. Blocking intestinal bacterial enzymatic functions may also improve intestinal homeostasis and improve efficacy and decrease toxicity of conventional and developing IBD therapies, as have been accomplished for cancer therapies (Pharmacomicrobiomics) [143].

Fully understanding the interactions between microbiota and the host immune system, in concert with environmental and genetic factors unique to each individual, is necessary to target the most effective therapies for each patient. Personalized diagnostic profiles will require identifying an individual’s metabolic functions and dominant microbial antigens by shotgun metagenomic and metabolomic profiling, in concert with host microbial transcriptomic and genetic profiling. Microbiota reciprocally interacts with each other and the diet to provide immunological signals to host and the same microbe sometimes behaves differently in different individuals [1, 11, 144]. Host genetic and nutritional factors will need to be considered in an integrated and personalized manner to increase the effectiveness and efficacy of microbiota-based therapies [111, 145]. Selection of optimal approaches and therapeutic targets based on analysis of an individual’s microbiota pattern will be important to replace missing or dysfunctional bacterial components. We believe that a combined strategy to promote homeostatic immune responses, improve mucosal barrier function and restore eubiosis by targeting dominant pathobionts and replacing missing protective species or their functions by manipulating the bacterial microbiota and diet may be best. This integrated approach should provide a more physiologic, safer and more cost-effective means to sustained remission of IBD than the current lifelong treatments with immunosuppressives. It is our belief that this approach will be more effective as maintenance therapies once induction of disease remission has been accomplished by traditional therapies, but then toxic induction regimens can be withdrawn to decrease toxicity.

## Conclusions and a path to improve personalized treatment

**Fig. 2:**
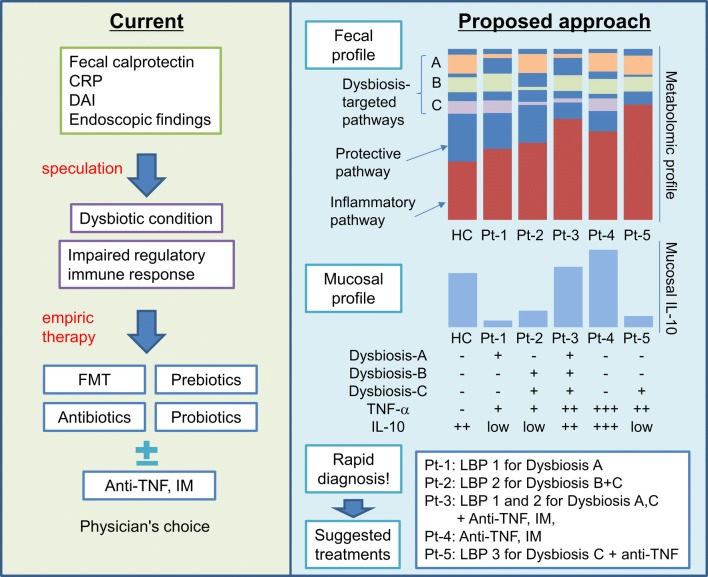
Current and proposed treatment strategies in microbe-based treatment for IBD. Currently, we diagnose and treat IBD patients based on clinical parameters including fecal calprotectin, serum CRP level, disease activity index (DAI) and endoscopic findings. These clinical observations do not provide insight into the degree of mucosal dysbiosis or impaired regulatory immune response in IBD patients. Therefore, empiric microbe-based therapies are used, such as existing probiotics, prebiotics, antibiotics, fecal microbial transplantation in addition to standard of care anti-TNF agents or immunomodulators (IM). Since these empiric treatments have a limited efficacy in current clinical practice, we propose a more rational and scientific approach based on the fecal microbial and mucosal immune profiles in each IBD patient determined by rapid diagnosis tests. These fecal metabolic profiles and mucosal immune cytokine expression levels allow us to provide more effective and lower toxic microbe-based treatments based on various combinations of protective bacterial strains (*LBP* live biotherapeutic products) that are then applied in a customized way to restore microbial homeostasis based on dysbiosis in an individual patient. This approach can potentially provide cost-effective, nontoxic treatment and higher quality of life for IBD patients. *HC* healthy control, *Pt* IBD patient

Human IBD includes genetically and clinically heterozygous patient subpopulations with very unique intestinal bacterial compositions and functions that help determine immune responses and disease outcomes. Therefore, we believe that it will be feasible to evaluate the microbe/immune profiles by rapid diagnostic tests of microbiota functional and mucosal immune profiles to direct highly effective and safe treatments in a personalized manner (Fig. 2). Restoring impaired regulatory immune cell activity by correcting dysbiosis and defective microbial metabolic functions is a novel and highly promising therapeutic approach to managing IBD in a more physiologic, safer and sustained manner. Unveiling the mechanisms underlying specific defective bacteria–host interactions in each IBD patient will enable precision editing of microbiota and their function with maximum effectiveness and efficiency.
